# Matriptase cleaves the amyloid-beta peptide 1–42 at Arg-5, Lys-16, and Lys-28

**DOI:** 10.1186/s13104-018-4040-z

**Published:** 2019-01-03

**Authors:** Li-Mei Chen, Karl X. Chai

**Affiliations:** Burnett School of Biomedical Sciences, University of Central Florida College of Medicine, 4000 Central Florida Boulevard, Bldg. 20, Rm. 323, Orlando, FL 32816-2364 USA

**Keywords:** Amyloid, Matriptase, Secretase

## Abstract

**Objective:**

The type-II transmembrane extracellular serine protease matriptase was shown to cleave at Arg-102 in the amino-terminal region of the amyloid precursor protein (APP). In this study we determined matriptase cleavage sites in the amyloid-beta (Aβ) peptide region of APP (Asp-597 to Ala-638 in the APP695 isoform). A recombinant human matriptase protease domain was used to cleave a synthetic human Aβ1–42 peptide. The human APP695 or mutants at the candidate matriptase cleavage sites was co-expressed with the human matriptase or its protease-dead mutant in HEK293 cells to evaluate matriptase cleavage of APP. Overexpression of matriptase in the M17 human neuroblastoma cells was performed to determine the effect of matriptase expression on endogenous APP.

**Results:**

The human Aβ1–42 peptide can be cleaved by the matriptase serine protease domain, at Arg-5, Lys-16, and Lys-28, as determined by matrix-assisted laser desorption ionization time-of-flight mass spectrometry. Co-expression of matriptase but not its protease-dead mutant with APP695 resulted in site-specific cleavages of the latter. Replacement of Arg-601 (Arg-5 in Aβ1–42) by Ala in APP695 prevented matriptase cleavage at this site. Overexpression of matriptase but not its protease-dead mutant in the M17 cells resulted in a significant reduction of the endogenous APP quantity.

## Introduction

The most recognizable pathologic feature of Alzheimer Disease (AD) is the amyloid plaques, formed by the amyloid-beta (Aβ) peptides [1]. Proteolytic cleavages of the amyloid precursor protein (APP) by β- and γ-secretases produce the Aβ peptides, of which Aβ1–40 is the most abundant and Aβ1–42 the most toxic [2]. APP cleavages by α- and γ-secretases release the non-toxic P3 peptide, precluding the production of Aβ1–40 or Aβ1–42.

Matriptase is a type-II single-transmembrane extracellular serine protease [3], capable of shedding the extracellular domain (ECD) of membrane proteins [4]. Recently, the shed-off soluble matriptase was shown to cleave the APP at Arg-102 in cultured neuroblastoma cells, an event accompanied by a reduced Aβ1–40 production [5]. We have previously mapped matriptase cleavage sites to the Aβ peptide region of APP at R5, K16, and K28 [6]. In this study we confirmed these matriptase cleavage sites using a synthetic human Aβ1–42 peptide and the serine protease domain of human matriptase. The R5 (Arg-601 in APP695) site was further confirmed by co-expressing an Arg-601/Ala mutant APP695 with human matriptase in HEK293 cells. Matriptase expression in the M17 human neuroblastoma cells resulted in reduction of the endogenous APP quantity.

## Main text

### Methods

#### Cleavage of Aβ1–42 by matriptase

A recombinant human matriptase protease domain (MatPD, Cat. No. 3946-SEB-010) and a synthetic human Aβ1–42 peptide (Cat. No. 1428/100U) were purchased from Bio-Techne (Minneapolis, MN). The human Aβ1–42 peptide (1 µg) was mixed with MatPD (0.1 µg or 0.3 µg) in a total volume of 20 µl with the reaction buffer (50 mM Tris–HCl, pH 8.5). Samples containing Aβ1–42 (1 µg) or MatPD (0.3 µg) alone were also prepared. The reactions were carried out at 37 °C for either overnight or 1.5 h, and the samples were analyzed by SDS-PAGE and stained with Coomassie Brilliant Blue. A duplicate sample containing 1 µg of Aβ1–42 and 0.3 µg of MatPD and one containing only 1 µg of Aβ1–42, both incubated for overnight, were sent for MALDI-TOF MS (matrix-assisted laser desorption ionization time-of-flight mass spectrometry) analysis, performed by the University of Florida Proteomics & Mass Spectrometry Core on a 4700 Proteomics Analyzer (Applied Biosystems, Foster City, CA).

#### Cleavage of APP in cultured cells by matriptase

Matriptase cleavage site mutants were generated using the QuickChange Site-Directed Mutagenesis Kit (Agilent Technologies, Inc., Santa Clara, CA) and an APP695-EGFP plasmid coding for the human APP695 fused with the enhanced green fluorescent protein (EGFP) on the carboxyl terminus of APP695. These plasmids were used for co-transfection of HEK293 cells with the cDNA coding for human matriptase, or a protease-dead matriptase mutant (S805A), or the empty vector (pLVX-Puro, Clontech Laboratories, Inc., Mountain View, CA), as described previously [4]. Alternatively the co-transfection was performed with the cDNA coding for the mouse TMPRSS6 (matriptase-2) [4]. The M17 human neuroblastoma cells were cultured and transfected as described previously [4]. At 24 h after the transfection, cells were lysed in RIPA buffer and the lysate was analyzed by SDS-PAGE and western blotting for various target proteins (matriptase, β-tubulin, or GAPDH) as described previously [4]. For detection of the APP695-EGFP fusion protein an EGFP monoclonal antibody (4B10, Cell Signaling Technology, Danvers, MA) was used. For detection of the endogenous APP in the M17 cells, a mouse monoclonal β-amyloid antibody (D-11) (Santa Cruz Biotechnology, CA) was used. Chemiluminescent visualization was performed on a ChemiDoc MP Imaging System (Bio-Rad Laboratories, Hercules, CA). Densitometry was performed using the Image Lab Software (Version 6.0.0, Bio-Rad Laboratories) for the quantitation of native APP or GAPDH levels in the M17 cells. Student’s *t* test (one-tailed) was applied to evaluate the means between paired sample groups from three independent experiments.

### Results

#### Cleavage of Aβ1–42 by matriptase

**Fig. 1:**
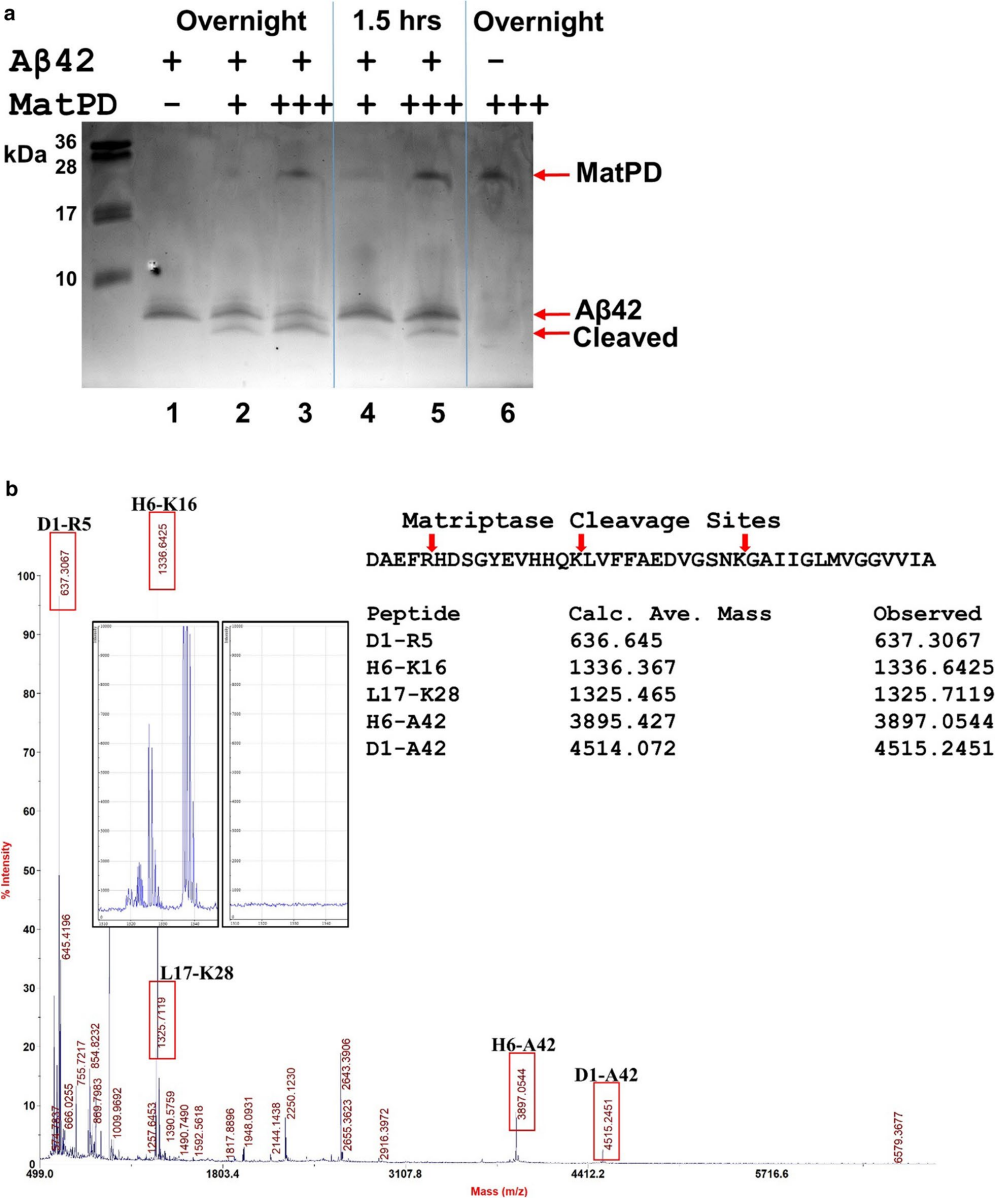
Cleavage of Aβ1–42 by matriptase. **a** Synthetic human Aβ42 (1 µg, Bio-Techne) was mixed with a recombinant human matriptase protease domain (MatPD, “+” = 0.1 µg, “+++” = 0.3 µg, Bio-Techne) in a total volume of 20 µl with reaction buffer. Samples containing Aβ42 or MatPD alone were also prepared. The reactions were carried out at 37 °C for either overnight or 1.5 h, as indicated, before the samples were analyzed by SDS-PAGE and stained with Coomassie Brilliant Blue. **b** Duplicate samples as described for lanes 1 and 3 were sent for MALDI-TOF MS analysis, performed by the University of Florida Proteomics & Mass Spectrometry Core on a 4700 Proteomics Analyzer (Applied Biosystems). Peptide fragments generated by MatPD from Aβ42 were identified in the data graph from Sample 3 (Aβ42 + MatPD), as shown by the red boxes with the calculated (average) and observed masses listed in the table. Insets: left—separation of peaks “L17-K28” and “H6-K16” by MSight [21]; right—spectrum from Sample 1 (Aβ42 alone) set to the same intensity scale and in the same mass range. Other boxed peaks were also examined by MSight over the corresponding mass spectrum ranges between Samples 3 and 1 (not shown)

As shown in Fig. 1a, matriptase was readily able to cleave the synthetic human Aβ1–42, producing fragments of lower molecular mass seen in the SDS-PAGE. MALDI-TOF MS confirmed the cleavage sites at R5, K16, and K28, as shown in Fig. 1b, with a good agreement of the observed molecular mass of the cleaved peptide fragments to the corresponding calculated average molecular mass.

#### Cleavage of APP in cultured cells by matriptase

**Fig. 2:**
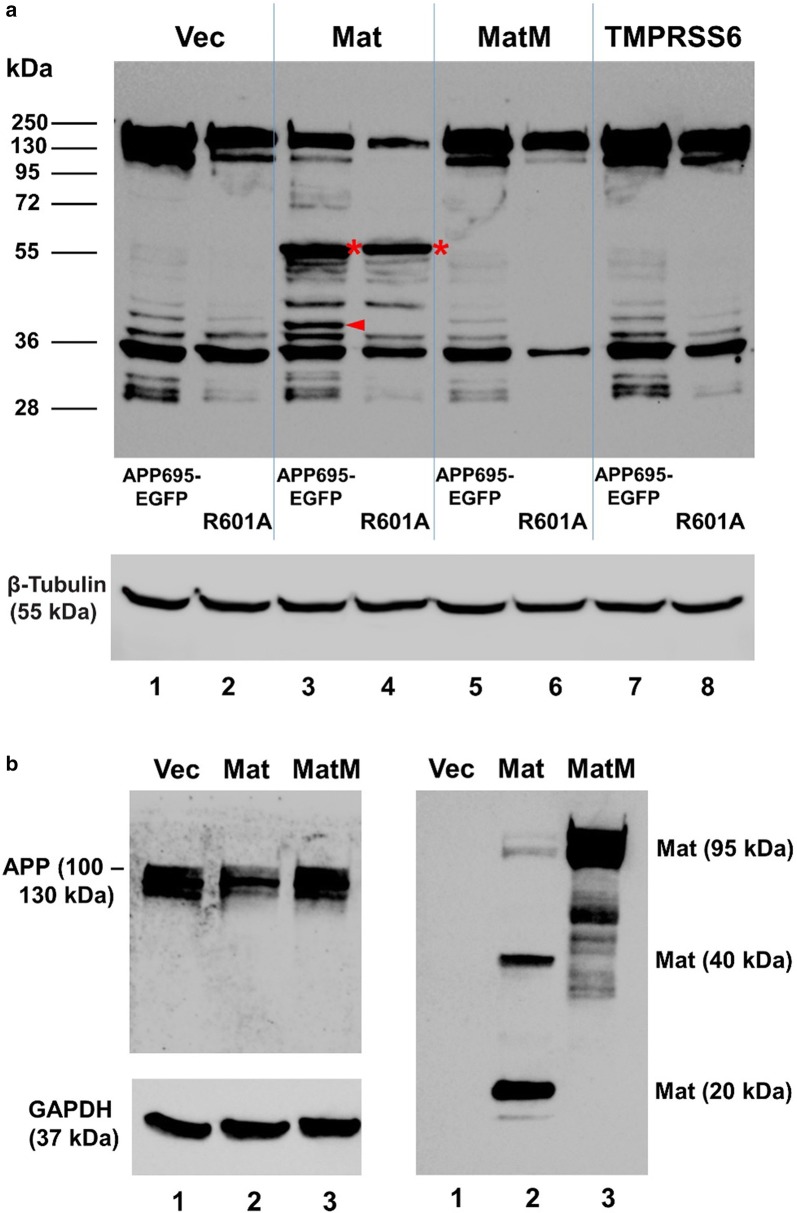
Cleavage of APP in cultured cells by matriptase. **a** The APP695-EGFP (lanes 1, 3, 5, and 7) or the APP-R601A-EGFP mutant plasmid (R601A, lanes 2, 4, 6, and 8) (0.6 µg for each *per* transfection) was co-transfected in HEK293 cells with an empty plasmid (pLVX-Puro) (Vec, lanes 1 and 2), or the cDNA coding for human matriptase (Mat, lanes 3 and 4), protease-dead matriptase (MatM, lanes 5 and 6), or mouse TMPRSS6 (lanes 7 and 8) (0.4 µg for each *per* transfection). Twenty micrograms of total cell lysate from each sample were resolved on SDS-PAGE and western-blotted with an EGFP antibody or a β-tubulin antibody. The 55-kDa matriptase-specific APP-EGFP CTF is marked by the red asterisk, and the 38-kDa matriptase-specific APP-EGFP CTF is marked by the red arrowhead. **b** An empty plasmid (pLVX-Puro) (Vec, lane 1), or the cDNA coding for human matriptase (Mat, lane 2) or protease-dead matriptase (MatM, lane 3) (1.5 µg for each *per* transfection) was transfected in M17 human neuroblastoma cells. Twenty micrograms of total cell lysate from each sample were resolved on SDS-PAGE and western-blotted with an APP antibody, a matriptase antibody, or a GAPDH antibody. The data shown here were representative from three independently repeated experiments

A co-expression of the wild-type human matriptase (Mat) with APP695-EGFP in the HEK293 cells resulted in matriptase-specific cleavages of the APP695 ECD. A major APP-EGFP CTF (carboxyl-terminal fragment) at 55 kDa (marked by the red asterisk) and a major APP-EGFP CTF at approximately 38 kDa (marked by the red arrowhead) were detected by the EGFP antibody in the cells expressing the wild-type matriptase (Fig. 2a, lane 3). Co-expression of APP695-EGFP with the protease-dead matriptase (MatM) however, was not associated with these matriptase-specific APP-EGFP CTFs (Fig. 2a, lane 5). The 38-kDa CTF was identified as the potential matriptase cleavage product at Arg-601, which would contain an APP CTF with a calculated molecular weight of 10.5 kDa and the fused EGFP, at 27 kDa. The candidate matriptase cleavage site Arg-601 (R5 in Aβ1–42) in APP695-EGFP was replaced with Ala in the mutant APP-R601A-EGFP. The 38-kDa CTF was no longer observed when APP-R601A-EGFP was co-expressed with matriptase but this site-specific mutation did not affect the cleavage that produced the 55-kDa CTF (Fig. 2a, lane 4). The site for producing the 55-kDa APP-EGFP CTF would map in a region further up from the Aβ-peptide region toward the APP amino terminus. The Arg-601-specific 38-kDa and the 55-kDa CTFs produced by the co-expression of matriptase were not observed with the co-expression of TMPRSS6 (matriptase-2), a closely related type-II transmembrane extracellular serine protease (Fig. 2a, lane 7).

In Fig. 2b, we show that the wild-type matriptase (Mat) expressed in the M17 cells was auto-activated, displaying the characteristic lower molecular weight fragments (right panel, lane 2), as described previously [7]. Matriptase auto-activation requires its catalytic triad [7], thus the protease-dead matriptase (MatM) was unable to undergo auto-activation and remained mainly in the 95-kDa form (right panel, lane 3). Associated with the expression of the active matriptase in the M17 cells, the endogenously expressed APP, observed at 100–130 kDa as previously reported for these cells [8], was significantly reduced in quantity (left panel, lane 2). The APP level in the matriptase-expressing cells was determined to be at 0.65 ± 0.029 (*p* < 0.05) of that in the vector control (left panel, lane 1), with an average reduction of 35%. The APP level in the cells expressing the mutant matriptase was not changed when compared to the vector control (*p* > 0.05). The levels of GAPDH between the different cells also remained unchanged (*p* > 0.05). The epitope of the antibody used to detect the endogenous APP in the M17 cells maps to amino acid residues 663–687 in the APP ECD (588–612 in APP695) and did not allow for detection of any CTF resulting from the matriptase cleavages. The endogenous APP identified by this antibody represents the total cellular full-length APP.

### Discussion

The co-expression of human APP695 in the HEK293 cells with the wild-type human matriptase resulted in matriptase-specific cleavages, producing APP CTFs not observed with the co-expression of TMPRSS6 (matriptase-2), a closely related tryptic serine protease previously shown to cleave membrane receptor ECDs in the same cells [4]. We have determined a matriptase-specific cleavage site in the Aβ peptide region of APP, at Arg-601 (APP695 numbering). This site can be cleaved specifically by matriptase in APP when co-expressed in cultured cells, and in the Aβ1–42 peptide (R5) by the recombinant human matriptase protease domain. The α-secretase cleavage site K16 was also cleaved by matriptase in Aβ1–42 but we were unable to unequivocally demonstrate a matriptase-specific cleavage of this site (Lys-612) in APP695 due to the actions of the endogenous α-secretase. A site-specific mutation at Lys-612 would have prevented both the matriptase-associated cleavage at this site and also any α-secretase cleavage. An assignment of cleavage at K612 to matriptase in a cell with an endogenous α-secretase was therefore, not possible. We did not attempt to confirm the K28 site for matriptase cleavage in APP695 (Lys-624) by co-expressing a site-specific mutant in the HEK293 cells because we were unable to unequivocally identify an APP695 CTF generated by a matriptase cleavage at this site. The K28 site may only be accessible to matriptase in the Aβ peptides. The native, endogenously expressed APP in the M17 human neuroblastoma cells, which are a relevant model of APP processing in AD [8], can be quantitatively reduced when the cells expressed an active recombinant matriptase (Fig. 2b). We have previously shown that matriptase can act at the plasma membrane to cleave the Her2 ECD [4]. Thus matriptase may serve as an α-secretase. But it will require further experimentation to show matriptase cleavage of Lys-612 in the APP in a cell devoid of all other α-secretases to establish this.

The cleavage sites of R5, K16, and K28 are novel to matriptase as an Aβ-modifying protease, but the site-specific cleavage products had long been reported in AD patients. A single cut of Aβ1–42 at R5 by matriptase would produce Aβ6–42. In the amyloid deposits from the brains of 10 sporadic AD patients, 2 were shown to contain the variant Aβ6–42 [9]. The Aβ6–42 variant, along with Aβ6–40, was also found in the brains of AD patients with accompanying cerebral amyloid angiopathy [10]. In the human cerebrospinal fluid, SDS-stable non-covalent trimers of Aβ6–42 could be detected [11]. In 5 AD brains with high contents of Aβ, all were shown to contain the variants Aβ29–40 and Aβ29–43 at significant portions [12]. These variants would be produced by a cleavage at K28, which is a matriptase cutting site. Thus the matriptase cleavages of Aβ peptides may be considered relevant to AD.

Equally important is the question of whether matriptase is functionally expressed in the relevant part of the brain. One group who reported APP cleavage by matriptase failed to detect the matriptase protein in adult or elderly human brain and the reported RNAseq matriptase transcripts in human brain tissues were at a very low level [5]. However, neurons derived from human induced pluripotent stem cells expressed an abundance of the matriptase protein following 6 weeks of neuronal differentiation [5], while matriptase was implicated for a role in promoting neuronal differentiation from neural progenitor/stem cells [13] and in endothelial cell adhesion [14]. Moreover, matriptase has recently been reported to be upregulated among the most upregulated genes in the activated microglia when associated with plaques in the 5× FAD transgenic mice modeling the human AD pathology [15]. Microglia have a protective role against AD progression with their abilities to clear Aβ peptides, in the soluble, lipoprotein-associated, or fibril form and to wrap around the plaques preventing further Aβ fibrillization [16]. An upregulated functional matriptase presence in this context could be a facilitating factor toward such a protective role.

Before we showed matriptase as an Aβ peptide-cleaving protease, other proteases of different classes have been reported to do so, as well. The homologue of BACE1 (β-site amyloid precursor protein cleaving enzyme 1, an aspartic protease), BACE2 cleaves at F19 and F20 [17]. The secreted trypsin-like HtrA1 serine protease also cleaves the Aβ peptides, at V12 and Q15, and its protein is co-localized in amyloid deposits in AD human brain by immunohistochemistry [18]. HtrA1 is capable of disintegrating tau fibrils and Aβ1–42 aggregates in vitro via a protease-independent mechanism [19]. A number of metalloproteases are capable of extracellular degradation of Aβ, these include ACE (angiotensin-converting enzyme), IDE (insulin-degrading enzyme), MMP-9 (matrix metalloprotease-9), and the most potent of the class, NEP (neprilysin) [20]. From this study we add matriptase to the group of extracellular proteases capable of cleaving the Aβ peptides.

### Limitations

- In this study we identified matriptase as an Aβ peptide-processing protease and determined its cleavage sites in Aβ1–42. At least one site was shown to be cleaved by matriptase in the APP in cultured cells and matriptase expression could reduce endogenously expressed APP in a cell type originating from the central nervous system. The study however, is limited with only in vitro experiments while the potential interactions between matriptase and APP or Aβ peptides in the in vivo physiological context remains to be investigated.

## Abbreviations

Aβ: amyloid-beta peptides
ACE: angiotensin-converting enzyme
AD: Alzheimer disease
APP: amyloid precursor protein
BACE1: β-site amyloid precursor protein cleaving enzyme 1
CTF: carboxyl-terminal fragment
ECD: extracellular domain
EGFP: enhanced green fluorescent protein
FAD: familial Alzheimer disease
GAPDH: glyceraldehyde 3-phosphate dehydrogenase
IDE: insulin-degrading enzyme
MALDI-TOF MS: matrix-assisted laser desorption ionization time-of-flight mass spectrometry
MatPD: recombinant human matriptase protease domain
MMP-9: matrix metalloprotease-9
NEP: neprilysin
SDS-PAGE: sodium dodecyl sulfate–polyacrylamide gel electrophoresis
